# Contrasting Impacts of Ubiquitin Overexpression on Arabidopsis Growth and Development

**DOI:** 10.3390/plants13111485

**Published:** 2024-05-28

**Authors:** Peifeng Yu, Zhenyu Gao, Zhihua Hua

**Affiliations:** 1 Department of Environmental and Plant Biology, Ohio University, Athens, OH 45701, USA; py989117@ohio.edu (P.Y.); gaozhenyu@caas.cn (Z.G.); 2 Interdisciplinary Program in Molecular and Cellular Biology, Ohio University, Athens, OH 45701, USA; 3 State Key Laboratory of Rice Biology and Breeding, China National Rice Research Institute, Hangzhou 310006, China

**Keywords:** growth and development, ubiquitin, overexpression, the 26S proteasome, seed yield

## Abstract

In plants, the ubiquitin (Ub)-26S proteasome system (UPS) regulates numerous biological functions by selectively targeting proteins for ubiquitylation and degradation. However, the regulation of Ub itself on plant growth and development remains unclear. To demonstrate a possible impact of Ub supply, as seen in animals and flies, we carefully analyzed the growth and developmental phenotypes of two different *poly-Ub* (*UBQ*) gene overexpression plants of *Arabidopsis thaliana*. One is transformed with *hexa-6His-UBQ* (designated *6HU*), driven by the cauliflower mosaic virus *35S* promoter, while the other expresses *hexa-6His-TEV-UBQ* (designated *6HTU*), driven by the endogenous promoter of *UBQ10*. We discovered that *6HU* and *6HTU* had contrasting seed yields. Compared to wildtype (WT), the former exhibited a reduced seed yield, while the latter showed an increased seed production that was attributed to enhanced growth vigor and an elevated silique number per plant. However, reduced seed sizes were common in both *6HU* and *6HTU*. Differences in the activity and size of the 26S proteasome assemblies in the two transgenic plants were also notable in comparison with WT, suggestive of a contributory role of *UBQ* expression in proteasome assembly and function. Collectively, our findings demonstrated that exogenous expression of recombinant Ub may optimize plant growth and development by influencing the UPS activities via structural variance, expression patterns, and abundance of free Ub supply.

## 1. Introduction

Ubiquitin (Ub) is a 76-amino acid small protein that is conserved and ubiquitously present in all eukaryotic cells [1]. It was first isolated and termed by Goldstein and co-workers in searching for thymic peptide hormones, and later confirmed by Wilkinson et al. as the same protein of ATP-dependent proteolysis factor 1 (APF-1) [2,3]. The identification of ATP-dependent proteolysis that requires APF-1 resulted in the Nobel Prize-winning discovery of ATP-dependent and Ub-mediated protein degradation [4,5,6]. To date, Ub has been recognized as a post-translational modifier that rivals the phosphate group for covalent conjugation with nearly all intracellular proteins in a certain stage of their lifespan [7,8,9]. Similar to phosphorylation, the process of protein modification by Ub is termed ubiquitylation. It starts with an ATP-dependent activation of free Ub moieties by one E1 Ub-activating enzyme, proceeds by conjugating the activated Ubs with a small family of E2 Ub-conjugating enzymes, and ends by covalently linking the Ub from the E2~Ub conjugates (“~” indicates a high-energetic thioester bond) to ubiquitylation substrates that are specifically recognized by a large group of E3 Ub ligases [10]. Iterative ubiquitylation can form a poly-Ub chain on a ubiquitylation site of the substrate. Depending on the types of ubiquitylation, such as monoubiquitylation versus polyubiquitylation, and the topology of poly-Ub chains, ubiquitylated proteins can be targeted for degradation either in the 26S proteasome complex or by autophagy if they are associated with protein aggregates or damaged organelles. In addition, ubiquitylated proteins can also go through non-proteolytic processes, such as changes in their cellular localization, membrane trafficking, and activities [9].

The vast outcomes and a large pool of substrates of ubiquitylation led numerous studies, including plant biology research, to focus on the proteomic identification of ubiquitylated proteins and functional characterization of the ubiquitylation pathways whose specificities are determined by the E3 ligases in the past two decades (see [7]). All these studies sought to discover which substrates and how Ub is attached, i.e., the regulation of the ubiquitylated proteome (ubiquitylome). While the ubiquitylome may directly impact cellular growth and development, the homeostasis of Ub also has a profound influence through limiting the supply of free Ubs. Several studies in non-plant biology research have demonstrated a stress-regulated *Ub* gene expression and adverse impacts of down- and up-regulation of Ub supply on cellular function and individual survival. Multiple stressors, such as heat, starvation, oxidative stresses, and DNA-damaging agents, were shown to increase the expression of *poly-Ub* genes in yeast [11], mammalian cells [12,13], mouse epidermal tumors [14], and atrophying rat muscles [15], presumably to assist in the clearance of damaged and toxic proteins induced by stresses.

While it is apparent that downregulation of *Ub* gene expression is destructive by limiting the free Ub supply, exogenous overexpression of *Ub* can also disrupt normal growth and development in several organisms. In yeast, overexpression of *Ub* results in changes in stress tolerance but does not markedly alter the normal growth of cells [16]. In flies, upregulation of Ubs is toxic to pupal development but, in contrast, increases the lifespan of adults, preferentially in males [17]. The mammalian nervous system also seems sensitive to *Ub* overexpression. It was shown that increasing Ub levels impaired learning, reduced synaptic plasticity, and promoted glutamate ionotropic receptor AMPA (GRIA) receptor degradation in mice in a dose-dependent manner [18]. The same research group also discovered that a moderate increase in Ubs improved neuromuscular junction (NMJ) function, while robust upregulation of *Ub* gene expression reduced muscle development and motor coordination [19]. These studies collectively implied a dosage-dependent and cell-type-specific influence of *Ub* overexpression.

Compared to mammals, the number of *Ub* genes increases dramatically in plant genomes via distinct duplication mechanisms [20]. While there are two *Ub-ribosomal fusion* (also known as *Ub-extension*) genes and two *poly-Ub* genes in mice and humans [21], four *Ub-extension* genes, five *poly-Ub* (designated *UBQ* hereafter) genes, and five *UBQ*-like genes, in total 14 *Ub* genes, were identified from early genomic studies in the model flowering plant, *Arabidopsis thaliana* (Arabidopsis hereafter) [22,23]. Through a comprehensive comparative genomic study and a deep genome reannotation by Closing Target Trimming [24], we discovered an Arabidopsis *Ub* gene family that comprises 77 members, whose protein products carry at least one ubiquitin domain (Pfam ID: PF00240). Among these, 16 are *UBQ* or *UBQ*-like genes carrying coding sequences for two or more conserved Ub repeats [20]. Although not all members of the *Ub* gene family encode a Ub moiety capable of being conjugated with ubiquitylation substrates due to sequence diversification, the large expansion of the *Ub* gene family and increasing number of *UBQ* or *UBQ*-like genes in Arabidopsis indicate a high demand of Ub supply in plants [20,25].

The large duplications also resulted in differential gene expression regulation of *UBQ* genes. For example, the expression of five Arabidopsis *UBQ* genes was discovered to be differentially regulated in different organs or in response to different environmental changes [25]. Among them, *UBQ10*, the most highly expressed *UBQ* in Arabidopsis, was discovered to express constitutively. Its constitutively high expression shaded the total *UBQ* mRNA changes in Arabidopsis seedlings upon heat-shock treatments, which seems contradictory to the heat stress response of *Ub* gene expression in other organisms, including in tobacco mesophyll protoplast-derived cultures and maize seedlings [26,27]. This discrepancy might result from (1) a high number of *UBQ* genes duplicated in Arabidopsis, (2) a need for different heat stress treatments, and/or (3) a lack of coordinated induction of *UBQ* genes upon the heat-shock treatments [25]. While it remains unknown whether heat treatment can upregulate Ub protein levels, this study suggests that a high expression of constitutively expressed *UBQ10* dominates the Ub supply in Arabidopsis, thus making only small alterations of total *UBQ* mRNA levels, although varying expression responses were observed for the other four mildly expressed *UBQ* genes [25].

Arabidopsis seedlings appear to tolerate *Ub* overexpression better than animals do. The first ubiquitylome was identified in yeast *Saccharomyces cerevisiae* for proteins that were conjugated with recombinant 6His-tagged Ubs [28]. Conjugation of 6His-tagged Ubs allows ubiquitylated proteins to be purified via nickel-nitrilotriacetic (Ni-NTA)-based affinity purification and identified by mass spectrometry (MS) using the diGly (Lys-ε-Gly-Gly) Ub footprint [28]. Using the same strategy, Saracco et al. developed a *hexa-6His-UBQ* Arabidopsis transgenic plant that expressed six head-to-tail linked *6His*-tagged *Ub* moieties driven by the cauliflower mosaic virus (CaMV) *35S* promoter (*HU* for a *6His*-tagged *Ub* moiety and *6HU* for the *hexa-6His-UBQ* transgene and transgenic plants, hereafter) [29]. Since multiple transgenic lines with high expression of *6HU* were discovered to be phenotypically normal under the multiple conditions tested, one stable transformant with high expression of *6HU* was chosen for the first comprehensive characterization of ubiquitylome in Arabidopsis [29]. This plant was further utilized for several advanced Arabidopsis ubiquitylome analyses, including the discoveries of a deep catalog of ubiquitylation substrates as well as the ubiquitylomes involved in pathogen response and photomorphogenesis [30,31,32].

While the *6HU* plant has generated a deep list of ubiquitylated proteins in Arabidopsis, the lack of biological influence of overproduced HU proteins is inconsistent with studies in yeast [16], flies [17], and mice [18,19]. In plants, the overexpressing wheat (*Triticum aestivum*) *Ta-Ub2* gene, which encodes a single Ub repeat, was shown to improve abiotic stress tolerance in tobacco and brachypodium [33,34]. Constitutive overexpression of *Ta-Ub2* driven by the CaMV *35S* promoter can also lead to slight growth inhibition of brachypodium under a normal growth condition [34]. Except for the study of *Ta-Ub2*, how the expression of *poly-Ub* genes impacts plant growth and development remains unclear. To address this question, we carefully re-examined the growth and reproductive phenotypes of the same *6HU* plant. For comparison, we also analyzed the growth and development of two *hexa-6His-TEV-UBQ* transgenic plants that we previously generated for expressing six head-to-tail linked *6His*- and *Tobacco Etch Virus protease cleavage site (TEV)*-tagged *Ub* moieties driven by the endogenous *UBQ10* promoter (*HTU* for a *6His-TEV*-tagged *Ub* moiety and *6HTU* for the *hexa-6His-TEV-UBQ* transgene and transgenic plants, hereafter) [20]. Our studies discovered that multiple factors, such as structure, abundance, and spatiotemporal expression patterns, contributed to the impact of exogenously expressed recombinant Ub on Arabidopsis growth and development.

## 2. Methodology

### 2.1. Plant Materials and Growth

The Arabidopsis reference accession Col-0 was used as the wildtype (WT) control. Seeds were vapor-phase surface-sterilized for 5 h in a sealed desiccator, with an open beaker containing 100 mL of NaOCl supplemented with 3 mL of HCl. After dark stratification in water at 4 °C for three days, seeds were germinated on half-strength Murashige–Skoog (1/2 MS) medium (Caisson Labs, Smithfield, UT, USA) with 1% (*w*/*v*) sucrose and 0.7% (*w*/*v*) agar. Seven-day (d)-old seedlings were then plotted in mixed soil containing 1/3 vermiculite, 1/3 peat moss, and 1/3 leaf compost and topsoil mix. Unless otherwise described, seedlings on plates or plants on soil were grown under a long-day (LD) photoperiod, with 16 h light (125 μmol m^−2^ s^−1^) and 8 h darkness, at 21 °C and 19 °C, respectively.

### 2.2. Phenotypic Analysis

For each genotype, ripened seeds were harvested and pooled from dried siliques. Before measurement, seeds were completely dried out for 3 days in a 37 °C incubator and acclimated at room temperature for at least 2 days. Photographs of 100 seeds from each of WT, 6*HU*, and 6*HTU* were captured under a Nikon SMZ1500 stereomicroscope (Nikon Corporation, Tokyo, Japan). The length, width, and area of individual seeds were measured and quantified using ImageJ (NIH, Bethesda, MD, USA) [35]. All the measurements were statistically analyzed using the Student’s *t*-test. For cotyledon area analysis, synchronized seeds were vapor-phase surface-sterilized, stratified, and germinated on 1/2 MS medium with 1% (*w*/*v*) sucrose and 0.7% (*w*/*v*) agar. Upon full expansion of cotyledons at 7 days after germination under a LD photoperiod, open cotyledons were photographed and quantified using ImageJ [35]. For silique clearing, ripened siliques randomly selected from primary inflorescences were immersed in 0.2 N NaOH and 1% SDS solution and incubated at room temperature for 3 days, before photographing under a Nikon SMZ1500 stereomicroscope.

### 2.3. Protein Immunoblotting Analysis

Tissues (100 mg for each sample) were harvested, flash-frozen in liquid nitrogen, and temporarily stored in a −80 °C freezer before analysis. Prior to protein extraction, the frozen tissues were pulverized on a cryo block using a GenoGrinder (SPEX SamplePrep, Metuchen, NJ, USA) at a temperature below 0 °C. Except for proteasome activity and fractionation assays, total protein was directly extracted from pulverized tissues by boiling in 2 × SDS sample buffer for 5 min. Total protein extract (20 µg per sample) was resolved in 10% or 6–20% gradient SDS-PAGE and blotted on polyvinylidene difluoride membranes (Immobilon-P; Millipore Sigma, Burlington, MA, USA) before immunoblotting analysis. Anti-β-Actin (1:5000 dilution), Anti-Ub (1:1000), and Anti-6His (1:1000) were purchased from Proteintech (Zürich, Switzerland), Santa Cruz Biotechnology (Dallas, TX, USA), and MilliporeSigma (Burlington, MA, USA), respectively. Anti-PBA1 (1:5000), Anti-RPN1 (1:5000), and Anti-RPT2 antibodies (1:5000) were as previously described [36]. After immunoblotting with primary antibodies, the blot was incubated with horseradish peroxidase-conjugated goat anti-rabbit or goat anti-mouse secondary antibodies (SeraCare, Milford, MA, USA) and displayed with Super Signal West Pico Chemiluminescent Substrate or Super Signal West Femto Maximum Sensitivity Substrate (Thermo Fisher Scientific, Waltham, MA, USA). Chemiluminescent signals on each immunoblot were scanned using an Azure 600 Western Blot Imaging System (AZURE Biosystems, Dublin, CA, USA).

### 2.4. Proteasome Activity Assay

After extraction in lysis buffer (50 mM Tris–HCl, pH 7.5, 5 mM MgCl_2_, 1 mM Na_2_EDTA, and 10% [*v*/*v*] glycerol), 10 μg of total protein, as determined by a Bradford assay (Bio-Rad, Hercules, CA, USA), from each clarified extraction was used for the assay, as previously described [37,38]. Briefly, the same amount of protein extract from each sample, at a volume of 20 μL, was incubated for 20 min at 37 °C in 1 mL of assay buffer (50 mM Tris–HCl, pH 7.0, 2 mM MgCl_2_, 1 mM ATP, and 2 mM β-mercaptoethanol) containing 100 μM of N-succinyl-leucyl-leucyl-valyl-tyrosyl-7-amino-4-methylcoumarin (suc-LLVY-AMC, MilliporeSigma) in the presence or absence of 50 μM of the proteasome inhibitor, MG132. Upon incubation, the reaction was quenched by mixing with 1 mL of 80 mM sodium acetate (pH 4.3). The fluorescence resulting from the released AMC was recorded in a TKO 100 fluorometer (Hoefer Scientific Instruments, San Francisco, CA, USA) with an excitation wavelength of 365 nm and an emission wavelength of 460 nm. Three biological replicates, each with three technical replicates, were assayed.

### 2.5. Fractionation of Proteasome Complexes by Gradient Sedimentation

Seven-day-old LD-grown seedlings from WT, 6*HU*, and 6*HTU* were separately frozen in liquid nitrogen, pulverized, and ground in 1.25 volumes of extraction buffer (20 mM Tris-HCl, pH 7.5, 10% glycerol, 2 mM ATP, 5 mM MgCl_2_, 1 mM DTT, 10 mM phosphocreatine, and 1 mg/mL creatine phosphokinase). Protein extracts were filtered through two layers of cheesecloth and clarified through centrifugation at 30,000× *g* for 20 min. The resulting 200 μL clarified extract was then loaded on top of an 11 mL linear glycerol density gradient ranging from 10% to 40% (*v*/*v*) in sedimentation buffer (100 mM Tris–HCl pH 7.4, 0.15 M NaCl, 0.5 M MgCl_2_, 2 mM ATP, and 15–40% glycerol) for ultracentrifugation separation at 100,000× *g* for 18 h at 4 °C. Fractions (0.5 mL) collected through a Gilson FC203B fraction collector (Gilson, Middleton, WI, USA) were assayed for proteasome activity or subjected to immunoblot analysis, with three selected 26S proteasome subunit antibodies that represent 19S lid (RPN1), 19S base (RPT2), and the 20S core particle (PBA1) [39,40].

## 3. Results

### 3.1. Distinct Morphological Changes of 6HU and 6HTU

Both *6HU* and *6HTU* transgenes were similarly constructed, except that there was a *TEV* tag between *6His* and *Ub* in the *HTU* moiety, a *cMyc* tag was added at the 5′-end of *6HTU*, and *6HTU* was driven by the endogenous promoter of *UBQ10* (Figure S1a). To ensure that HTU was properly cleaved from the 6HTU poly-Ubs and conjugated with ubiquitylation substrates, we compared the total proteins that were conjugated with HTU and HU by anti-6His and anti-cMyc antibodies in seven-day-old seedlings under both normal and proteotoxic stress conditions. A smear band with high-molecular-weight species was detected by the anti-6His antibody in both transgenic plants but not in WT, and the treatment with the proteasome inhibitor MG132 dramatically enhanced the band intensity (Figure S1b). In addition, immunoblotting analysis with the anti-cMyc antibody also detected a smear band with high-molecular-weight species in *6HTU* seedlings, particularly under MG132 treatment, but not in other samples, nor a band with the expected 65 kDa size of 6HTU poly-Ubs (Figure S1c). Therefore, HTU, similar to HU, can be properly cleaved by deubiquitylating proteases from 6HTU, readily conjugated with ubiquitylation substrates, and in response to MG132 treatment. Considering the stronger signal detected in *6HU* than that in *6HTU* under normal and MG132 treatment conditions (Figure S1b), it can be inferred that the expression of *6HU* was higher than *6HTU*, which is consistent with the strong activity of the *35S* promoter in Arabidopsis seedlings [41].

**Figure 1:**
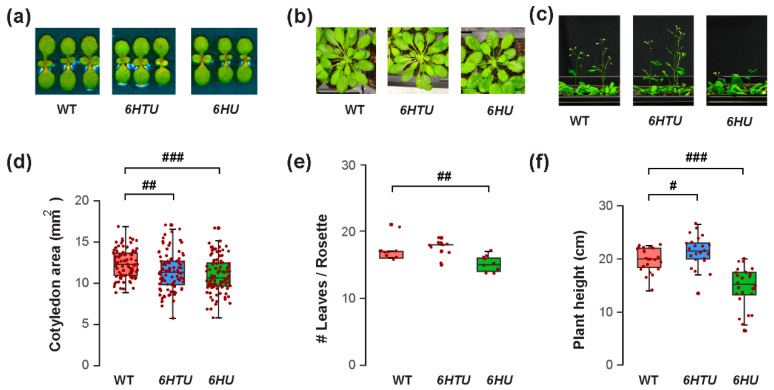
Exogenous expression of recombinant *UBQ* genes impacts vegetative growth and bolting differently. Synchronized seeds were germinated and grown under an LD photoperiod for the assays. (**a**) Representative images of reduced cotyledon areas of seven-day-old LD-grown seedlings from *6HU* and *6HTU* compared to WT. (**b**) Representative 50-day-old SD-grown plants, showing that a smaller number of rosette leaves developed in *6HU* than that in WT and *6HTU*. (**c**) Representative plants at the bolting stage identified an increasing and a reducing growth vigor of *6HTU* and *6HU*, respectively, compared to WT. (**d**–**f**) Quantitative comparisons of cotyledon areas (**d**), number of rosette leaves (**e**), and plant height (**f**) among the three indicated genotypes. The data points of replicates in each boxplot are indicated with maroon dots, as well as in other figures throughout the paper. Statistically significant differences were calculated using the Student’s *t*-test. #, *p* < 0.05; ##, *p* < 0.01; ###, *p* < 0.001.

The varying recombinant Ub levels produced in *6HTU* and *6HU* allowed us to compare the physiological impacts of Ub overexpression in Arabidopsis. We first evaluated their morphology changes in comparison with WT at three different developmental stages: early seedling, mature rosette, and bolting. Different to no morphological difference observed by Saracco et al. [29], we found significantly smaller cotyledon areas in both *6HTU* and *6HU* compared to WT, with *6HU* having the smallest cotyledons among the three genotypes (Figure 1a,d). In addition, *6HU* had a smaller number of open rosette leaves than WT and *6HTU* in 50-day-old plants grown in a short-day (SD) photoperiod (8 h light/16 h darkness at 21 °C and 19 °C, respectively), indicating its slow growth (Figure 1b,e). This slow growth was further evidenced by its reduced height in three-week-old LD-grown plants (Figure 1c,f) and delayed bolting in two-week-old LD-grown plants compared to WT (Figure S2). In contrast to a smaller cotyledon area than WT, *6HTU* demonstrated a higher growth vigor than WT after bolting. It grew taller and developed more siliques per plant than WT (Figure 1c,f; Figures S2 and S3a). In contrast, *6HU* had the least growth vigor among the three plants compared. It bolted late and developed fewer siliques per plant than WT and *6HTU* (Figure 1c,f; Figures S2 and S3a).

### 3.2. Exogenous Ub Expression Can Increase or Decrease Seed Yield

**Figure 2:**
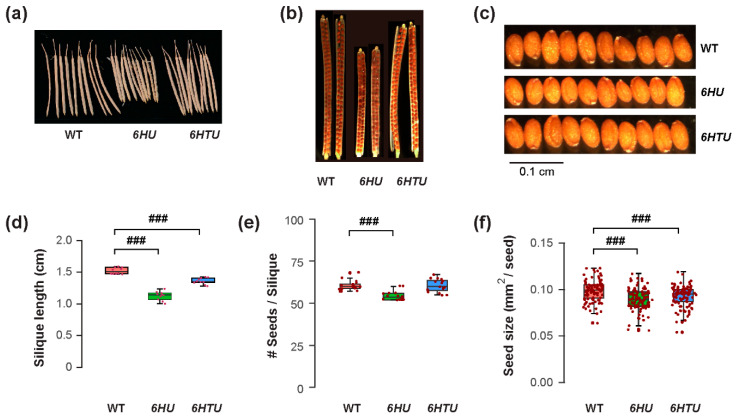
Exogenous expression of recombinant *UBQ* genes reduces silique and seed sizes. Plants were germinated and grown under the same condition until harvest for the assays. (**a**) Representative images showing shortened siliques that developed from primary inflorescences of *6HU* and *6HTU* plants compared to WT. Ten siliques from primary inflorescences were randomly selected from each genotype for photographing. (**b**) Cleared siliques with different numbers of seeds from WT, *6HU*, and *6HTU*. Ripened siliques from primary inflorescences were randomly selected and cleared in 0.2 N NaOH and 1% SDS solution for three days. Images were recorded using a Nikon SMZ1500 stereomicroscope. (**c**) Images showing that smaller sizes of matured seeds developed in *6HU* and *6HTU* than that in WT. Ten representative dried seeds were randomly selected and lined together for photographing from each genotype. (**d**–**f**) Quantitative comparisons of dried silique length (**d**), number of seeds per silique (**e**), and seed area (**f**) among the three indicated genotypes. The data points of replicates in each boxplot are indicated as in Figure 1. ###, *p* < 0.001 (Student’s *t*-test).

The differences in plant architecture and growth vigor indicated a possible opposite impact of ectopic *Ub* expression on reproduction, such as fruit development and seed production. Similar to smaller cotyledons in both *6HTU* and *6HU* compared to WT, both transgenic plants developed shorter siliques than WT, with the ones from *6HU* being the shortest (Figure 2a,d). When counting the number of seeds in each silique, we saw no difference between WT and *6HTU*, while *6HU* had a smaller number of seeds per silique than WT (Figure 2b,e). Careful examination of green siliques carrying mature green embryos eight days after anthesis (DAA) did not find obvious aborted seeds or unfertilized ovules in *6HTU* and *6HU*, suggesting that overexpression of these two transgenes did not impact fertilization. The differences in fruit size and seed number in each silique were consistent with seed size changes. Both *6HTU* and *6HU* had a smaller average seed size than WT (Figure 2c,f), which explains their smaller cotyledons in seven-day-old seedlings compared to WT (Figure 1a,d). Interestingly, we did not see obvious seed width changes among the three genotypes, but there were shorter seed lengths in *6HTU* and *6HU* than in WT, with *6HU* having the shortest average seed length (Figure S4).

Since seed yield is a very important agronomic trait, the changes in fruit and seed sizes and the number of siliques per plant may have direct impacts on seed yield per plant. To address this question, we carefully evaluated the seed production per plant among three genotypes. We observed a 1.2-fold higher average dry mass of seeds per plant produced in *6HTU* than that in WT. However, the average seed weight per plant of *6HU* reduced to 0.6-fold of that obtained in WT (Figure S3b). Therefore, similar to that found in mice [19], exogenous *Ub* expression can either promote or impede Arabidopsis growth and development.

### 3.3. Multiple Factors Contribute to the Influence of Ub Supply on Growth and Developmental Alterations in Arabidopsis

**Figure 3:**
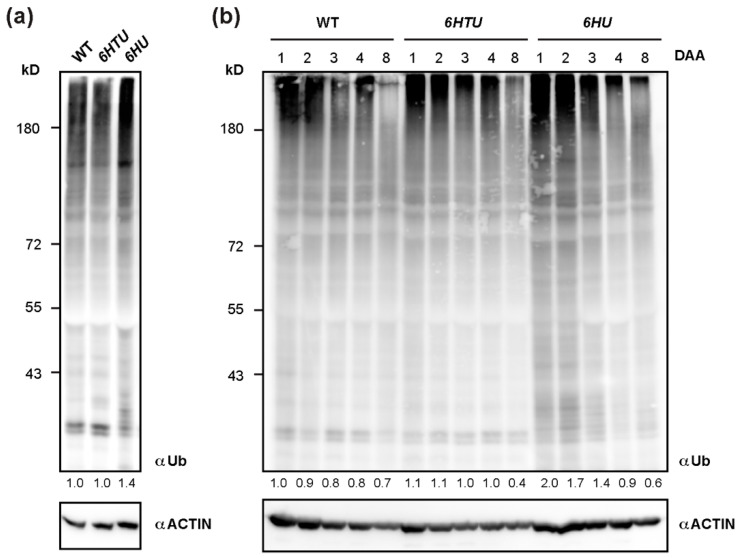
Dynamic comparison of total ubiquitylated proteins among WT, *6HU*, and *6HTU*. (**a**) One of the three independent immunoblotting analyses, showing that total ubiquitylated proteins from seven-day-old LD-grown seedlings were elevated in *6HU* but maintained a similar level in WT and *6HTU*. The number below each lane indicates the relative abundance of total ubiquitylated proteins of each sample that was first normalized as a ratio of its actin abundance and then to that of ubiquitylated proteins in WT. (**b**) Changes in total ubiquitylated proteins during early silique development in three genotypes highlighted a distinct and a similar dynamic regulation of protein ubiquitylation in *6HU* and *6HTU*, respectively, to that in WT. Siliques developed at 1, 2, 3, 4, and 8 DAA from primary inflorescences were harvested for protein immunoblotting analysis. The number below each lane was calculated as in (**a**).

To further confirm the upregulation of protein ubiquitylation in *6HTU* and *6HU*, we applied a Ub antibody to compare total proteins that are ubiquitylated by both native and recombinant Ub moieties. Since ubiquitylated proteins are vulnerable to post-lysis degradation, we extracted total protein from seven-day-old seedlings by directly boiling pulverized samples in 2 × SDS sample buffer. Upon three biologically independent analyses, we surprisingly found no significant difference in total ubiquitylated proteins detected by anti-Ub antibodies in WT and *6HTU* seedlings. However, a stronger smear band, which indicated total ubiquitylated proteins, was detected in *6HU* than in WT and *6HTU*, confirming overproduction of Ub supply in *6HU* (Figure 3a).

Since we previously demonstrated a dynamic decline of total ubiquitylated proteins in early silique development [36], the strong seed developmental phenotypes of *6HU* and *6HTU* led us to examine whether they had altered dynamics of ubiquitylated proteins, as compared to WT. After examining the total ubiquitylated proteins in siliques at 1, 2, 3, 4, and 8 DAA, we did not see a noticeable difference between *6HTU* and WT. However, much more ubiquitylated proteins, albeit in a declining trend, were detected in *6HU* siliques at 1, 2, and 3 DAA than those in *6HTU* and WT (Figure 3b). To rule out the possibility that the lack of dynamic differences in ubiquitylated proteins between *6HTU* and WT resulted from the absence of recombinant HTUs, we examined and detected proteins conjugated with HTU using the anti-6His antibody in the same set of protein samples. Interestingly, similar to the declining dynamics of total ubiquitylated proteins in all three plants, proteins conjugated with HTU and HU reduced upon silique development. However, the abundance of HTU-conjugated proteins declined more quickly than that of HU-conjugated proteins (Figure S5). Since the expressions of *6HTU* and *6HU* were driven by *UBQ10* and *35S* promoters, respectively, the dynamic difference in HTU- and HU-conjugated proteins further implied a transcriptional regulation of endogenous *UBQ* genes in silique development, as we discovered previously [36]. It was reported that the *35S* promoter was not active in immature embryos [42]. The strong HU-conjugates detected in immature siliques indicated that most of these products originated from maternal or non-embryonic tissues.

Considering the strong phenotypes seen in *6HU* and *6HTU* (Figure 1 and Figure 2; Figures S2–S4), the similar dynamics of total ubiquitylated proteins in seedlings and silique development between WT and *6HTU* suggested that the N-terminal 6His-TEV tag in HTU had an influence on plant growth and development. However, this influence could be further enhanced by increasing recombinant Ub supply in *6HU*. Since *6HTU* and *6HU* were driven by the *UBQ10* and *35S* promoters, respectively, the growth and developmental alternations between *6HTU* and *6HU* could also partially result from the differential expression patterns of the two transgenes. To address this question, we analyzed the plant height and silique length of a second independent *6HTU* transgenic plant, considering their significant changes in *6HTU* (Figure 1 and Figure 2). In three-day-old seedlings, *6HTU-7* yielded compatible recombinant Ub-conjugates, as observed in *6HU*, suggesting a higher production of Ub supplies than in *6HTU* (Figure S6a). However, *6HTU-7* possesses a similar growth vigor as *6HTU*, with no difference in height in three-week-old plants and a moderately but statistically significantly longer average length of ripened siliques than the latter (Figure S6b–e). Although we cannot rule out other growth and developmental differences between *6HTU* and *6HTU-7*, the similar plant heights at bolting and moderate changes in ripened siliques indicated their stronger growth vigor than *6HU*. Considering the positive effect of the N-terminal 6His-TEV tag in promoting *6HTU* growth, it is not likely that 6His-TEV and 6His tags in HTU and HU would result in opposite growth phenotypes in *6HTU-7* and *6HU*, respectively (Figure S6b). Therefore, multiple factors, such as the structure (*6HTU* vs. WT), abundance (*6HTU* vs. *6HTU-7*), and spatiotemporal expression patterns (*6HTU-7* vs. *6HU*) of recombinant Ubs, could impact Arabidopsis growth and development.

### 3.4. Both HU and HTU Increase Proteasomal Tolerance to MG132 Inhibition

**Figure 4:**
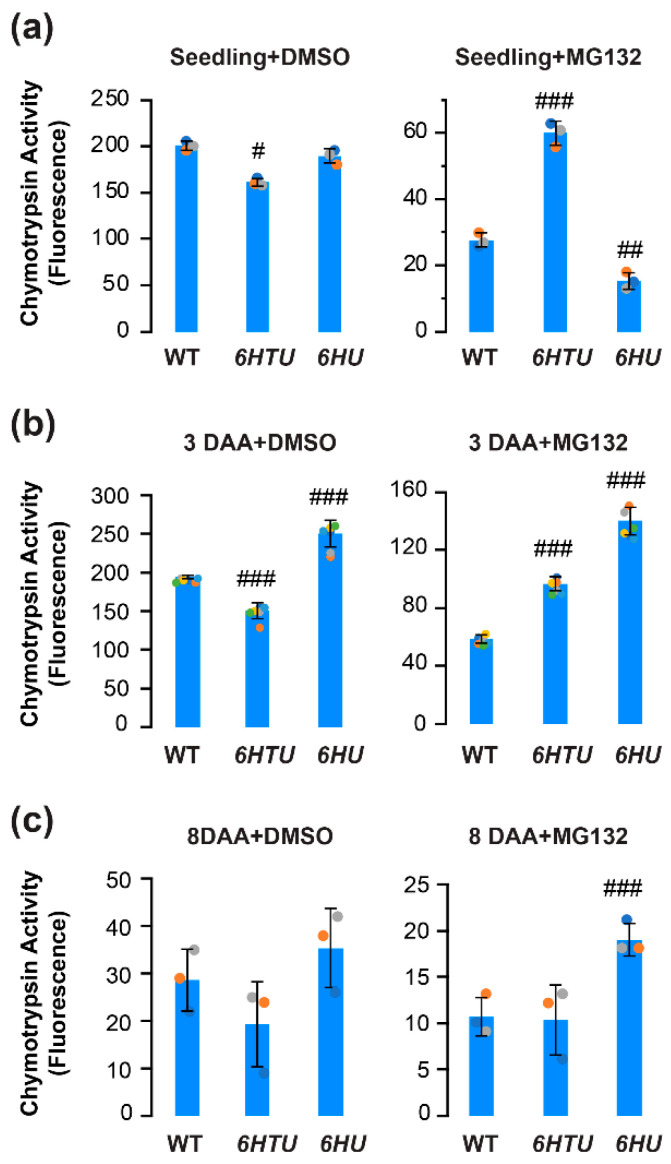
Comparison of in vitro proteasome activities indicated varying impacts of exogenous expression of recombinant *UBQ* genes on the function of the 26S proteasome. Total protein extracts were assayed for proteasome activities based on the fluoresce intensity of 7-amido-4-methylcoumarin (AMC) after cleavage from the substrate succinyl-leucyl-leucyl-valyltyroyl-7-amido-4-methylcoumarin (suc-LLVY-AMC). Bars represent the mean (±SD) of three biological replicates, which are indicated with green, orange, and cyan dots. (**a**) Proteasome activities in the extract of seven-day-old LD-grown seedlings from three indicated genotypes (left panel) and their different tolerances to MG132 inhibition (right panel). (**b**,**c**) Proteasome activities in the extract of 3-DAA (**b**) and 8-DAA (**c**) siliques from three indicated genotypes (left panel) and their different tolerances to MG132 inhibition (right panel). #, *p* < 0.05; ##, *p* < 0.01; ###, *p* < 0.001 (Student’s *t*-test).

Previous proteomics studies have revealed a heavily ubiquitylated proteasome in Arabidopsis seedlings [31,43]. The increased ubiquitylated proteins and different dynamics of HU-conjugated proteins led us to hypothesize an influence of Ub on proteasome activities. To demonstrate this hypothesis, we compared the differences in proteasome activities under both normal (DMSO) and proteotoxic (MG132) conditions in seven-day-old seedlings, immature (3-DAA, carrying heart embryos), and mature (8-DAA, carrying mature green embryos) siliques among WT, *6HTU*, and *6HU* (Figure 4; [36]).

Applying the same proteasome activity assay as we developed in our previous studies [36], we found a slight decline but a greater than 2-fold increase in seedling proteasome activity in *6HTU* under normal and proteotoxic conditions, respectively, in comparison with that in WT. In both conditions, the difference in seedling proteasome activities between *6HU* and WT was moderate. Although the proteasome activity in *6HU* seedlings seemed statistically more sensitive to MG132 inhibition than that in WT, it could be too low to be biologically significant (8% and 14% of their normal activities in *6HU* and WT, respectively; Figure 4a).

Similar to our previous studies [36], the WT proteasome activity declined slightly in 3-DAA siliques, reduced dramatically in 8-DAA siliques, but better tolerated MG132-mediated inhibition in both tissues than that in seven-day-old seedlings (Figure 4b,c). A similar trend was also evident for the proteasome activity in *6HTU* (Figure 4). However, the *6HU* proteasome activity in 3-DAA siliques was significantly higher than that in seven-day-old seedlings (Figure 4a,b). Consequently, *6HU* had significantly higher proteasome activity than WT in 3-DAA siliques under both normal and proteotoxic conditions. It also tolerated MG132 inhibition more strongly than WT and *6HTU* in 8-DAA siliques (Figure 4b,c). The higher tolerance to MG132 inhibition in *6HU* siliques suggested that ubiquitylated proteins prevent proteasomes from being inhibited by MG132. This protection could be obtained by both upregulation of ubiquitylated proteins and an extra tag at the N-terminus of Ub because *6HTU* is also more resistant to MG132 inhibition than WT in both seedlings and 3-DAA siliques (Figure 4a,b). However, a similar level of total ubiquitylated proteins was detected in *6HTU* and WT (Figure 3). Hence, we speculated that exogenous overexpression of HU and HTU may reduce the MG132 proteotoxic function through competitive binding with the proteasomes.

### 3.5. 6HU and 6HTU Have a Larger 26S Proteasome than WT

**Figure 5:**
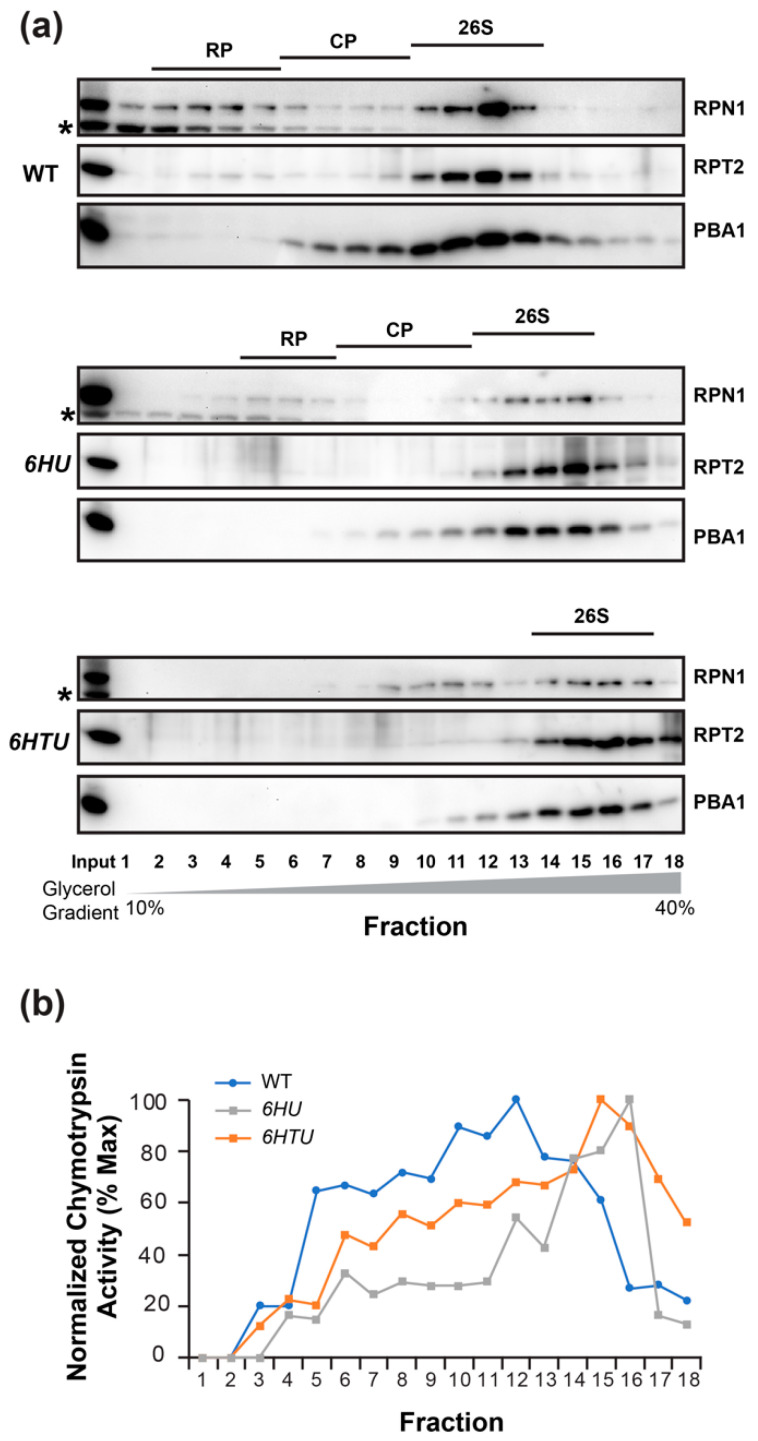
*6HU* and *6HTU* have a larger proteasome than WT. The 26S proteasomes from seven-day-old LD-grown seedlings were fractionated through a 10% to 40% glycerol gradient. The fractions possessing the holo-26S proteasome complexes are indicated by the presence of all three proteasome subunits in (**a**) or the normalized peak of chymotrypsin activity in (**b**). (**a**) Immunoblot detection of the three indicated proteasome subunits in different gradient fractions showed a shift of the holo-26S proteasome complexes to fractions with high glycerol concentrations in *6HU* and *6HTU* compared to WT. Asterisks indicate a non-specific protein species cross-reacted with RPN1 antibodies. (**b**) Chymotrypsin activity assay in different gradient fractions obtained from three indicated genotypes. The assay was performed as in Figure 4, except that the activity of each fraction was normalized to that obtained in the first fraction of each genotype.

If ubiquitylated proteins prevent MG132 inhibition through competitive interaction with the proteasome, we may see a larger 26S proteasome in both *6HU* and *6HTU* than in WT because HU and HTU have a larger mass than the endogenous Ub moieties (Figure S1a). The presence of epitope tags makes the Ub moieties conjugated with the proteasome, and the proteasome-associated ubiquitylated proteins a greater molecular mass than the endogenous Ubs. To test this hypothesis, we fractionated seven-day-old seedling proteins in a gradient of 10 to 40% glycerol solution. Based on the presence of the 3 represented subunits: 19S lid (regulatory particle non-ATPase 1, RPN1), 19S base (regulatory particle AAA-ATPase 2, RPT2), and 20S core (proteasome beta subunit A1, PBA1), in each of the 18 fractions, we were able to identify the fractions that possessed the regulatory particles (RP), the core proteases (CP), and the holo-complexes (26S) of the proteasomes in WT and *6HU*. We failed to identify the RP and CP fractions in *6HTU* but succeeded in finding the fractions with its 26S holo-complexes. Since the larger a protein complex is, the higher the glycerol concentration of the fraction in which it resides [40,43], the lower glycerol concentrations of fractions with the WT 26S proteasomes than those with the *6HU* and *6HTU* 26S proteasomes demonstrated that the latter two have a larger 26S proteasome than WT (Figure 5a). Considering the highest glycerol concentrations of fractions with the *6HTU* 26S proteasomes (Figure 5a), we predicted that the size of the 26S holo-complex would increase from WT, to *6HU*, to *6HTU*, which is consistent with the larger epitope tag present in HTU than that in HU (Figure S1a).

To confirm the presence of the 26S holo-complexes in fractions detected by the co-existence of RPN1, RPT2, and PBA1 (Figure 5a), we measured the proteasome activities in each protein fraction of WT, *6HU*, and *6HTU*. Since the 26S holo-complex has the highest chymotrypsin activity [40], the later peak appearance of normalized chymotrypsin activities in *6HTU* and *6HU* than that in WT further demonstrated a larger 26S proteasome in the former two plants than that in WT (Figure 5b).

## 4. Discussion

### 4.1. Challenges in Plant Ubiquitylome Studies

Proteins serve not only as intracellular workhorses in regulating metabolic pathways and signaling transduction but also as important cellular nutrient supplies during stresses, such as nitrogen and fixed carbon starvations. Hence, regulation of protein homeostasis can rapidly change growth and development from individual cells to organisms. Since the UPS is commonly recognized as the master regulator that determines the functional status of virtually all eukaryotic cellular proteins, many efforts have been invested in characterizing which and how proteins are ubiquitylated and degraded from yeast, to humans, to plants [7].

The milestone discovery of the diGly Ub footprint established the foundation for proteome-wide identifications of ubiquitylated proteins [28]. However, due to low stoichiometry, it is a critical step to enrich ubiquitylated proteins and/or ubiquitylation sites effectively before MS identification. To date, three affinity purification methods have been developed to purify ubiquitylated proteins or peptides on a proteome-wide scale [7]. The first applied epitope-tagged recombinant Ubs (e.g., HU) that were produced and conjugated with ubiquitylated proteins in vivo. The HU-conjugated ubiquitylated proteins can be enriched using Ni-NTA-based affinity purification [28]. The second utilized a strong affinity feature of tandem repeated Ub-binding entities (TUBEs) with poly-ubiquitylated proteins [44]. The third purified ubiquitylated peptides from trypsin-digested products of total protein using diGly Ub remnant antibodies [45]. Although the advancement of MS instrumentation has dramatically increased the sensitivity of proteome identification, all three affinity purification methods have limitations [7]. For example, TUBE cannot purify mono-ubiquitylated proteins. The diGly Ub remnant antibody enriches both ubiquitylated and neddylated proteins. In addition, it does not distinguish functional and non-functional ubiquitylated proteins. Ni-NTA-based purification of HU-conjugated ubiquitylation substrates has a high false discovery rate due to the presence of poly-His motifs in endogenous proteins, particularly in plant proteomes. To resolve these disadvantages, the first two methods have been applied in tandem to achieve cleaner ubiquitylomes in plants [29,30,31,32].

Our discovery of perturbation of HU and HTU conjugation to plant growth and development added a new challenge in plant ubiquitylome studies. The dramatic growth defects of the *6HU* plant complicated the interpretation of previous ubiquitylome data obtained through this plant [29,30,31,32]. Not only the low purity of Ni-NTA-enriched ubiquitylated proteins but also artificial ubiquitylation events by upregulated HU-conjugation could have resulted in a high rate of false discoveries. Hence, the ubiquitylome studies in plants need to be further improved.

### 4.2. Role of Protein Ubiquitylation in Seed Development

The reduced seed sizes and cotyledon areas in *6HU* and *6HTU* indicated a negative role of protein ubiquitylation in regulating seed size. Such negative impacts likely result in unwanted degradation of multiple positive regulators involved in seed development. Considering the integrative effects of embryo, endosperm, and the maternal integument layers on seed development and size control [46], multiple effectors are expected to be regulated through protein ubiquitylation in a temporal and spatial manner. For example, diminishing the function of Skp1-cullin (CUL) 1-F-box ubiquitin E3 ligase complexes in the double-knockout mutant for *Arabidopsis Skp1-like 1/2*, *ask1 ask2*, resulted in retarded and abnormal embryo development. The derived seeds either failed to germinate or germinated but were arrested to develop new root and leaf tissues [47]. Although the single *ask1* mutant in the Col-0 background has an extremely low fertility rate and retarded early embryogenesis, its seeds have a larger size than that produced in Col-0 WT plants, indicating an upregulation of nutrient remobilization from endosperm to embryo in late embryogenesis and seed maturation [48]. Protein ubiquitylation can also control seed sizes by impacting the proliferation of the maternal integument cells. Genetic screens and biochemical studies identified that a maternally produced ubiquitin receptor, DA1, regulates cell proliferation of integument tissues through physical interactions with two Really Interesting New Gene (RING)-type mono-subunit ubiquitin E3 ligases, DA2 and ENHANCER OF DA1 (EOD1)/BIG BROTHER (BB) [49,50]. Null mutants of *da1-1*, *da2-1*, and *eod1/bb* all yield large seeds and develop big sizes of other organs, indicating their negative roles in seed and organ size determination [50,51,52].

While it is not yet clear what ubiquitylation substrates are misregulated in the seed development of *6HU* and *6HTU*, our biochemical and expression analyses of the proteasome and autophagy activities in the early silique development uncovered a relay of these two systems in controlling the proteome homeostasis [36]. While the UPS is active in regulating cell division and differentiation in early seed development, upregulation of autophagy outcompetes the UPS in late embryogenesis toward maturation for nutrient remobilization, including proteasome recycling. We and others discovered that the UPS and the autophagy can reciprocally degrade the members of each system [36,37,53]. Therefore, their precise spatiotemporal regulation in seed development is essential for determining the seed’s size, weight, and viability. Considering the reduced silique and seed sizes of *6HU* and *6HTU* (Figure 2) and their upregulation of proteasome activities in 3- and 8-DAA siliques, particularly those from *6HU*, in comparison with WT (Figure 4), we speculated that the autophagy activities in both *6HU* and *6HTU* were compromised. It is worth further investigating the function of autophagy and stability changes in key autophagy members in these two plants, which may shed new light on the interplay of the UPS and autophagy in seed development.

### 4.3. Optimization of Ub Supplies for Enhancing Plant Growth Vigor and Seed Yield

The versatile regulatory roles of protein ubiquitylation offer great potential in optimizing plant growth and development and thus can be translated to improve crop production. The opposite functions of *6HU* and *6HTU* in improving Arabidopsis vegetative growth vigor (Figure 1) and seed yield (Figure 2; Figures S3 and S4) under a normal growth condition reflected that the endogenous UPS function was not optimized. Since the Ub supply itself can impact Arabidopsis growth and development through multiple factors in the structure, abundance, and spatiotemporal expression patterns of recombinant Ubs (Figure 1, Figure 2 and Figure 3 and Figure S5), we speculated that we may change the growth vigor and reproductive yield of plants, particularly crops, by optimizing the Ub supply in vivo through genetic engineering. In addition to previous studies showing increasing stress tolerance upon overexpression of a mono-Ub gene, *Ta-Ub2*, in tobacco and brachypodium [33,34], our study opens the door to a new biotechnology approach for improving crop production by manipulating the endogenous Ub supply.

## Data Availability

All relevant data can be found within the manuscript and its supporting materials.

## Supplementary Materials

The following supporting information can be downloaded at: https://www.mdpi.com/article/10.3390/plants13111485/s1.
