# Supplementary material for: Contrasting Impacts of Ubiquitin Overexpression on Arabidopsis Growth and Development
Source: Plants (Basel). 2024 May 28;13(11):1485. doi: 10.3390/plants13111485 (PMC11174952; doi:10.3390/plants13111485)
Supplement: Supplementary file 1 [file plants-13-01485-s001.zip › plants-2992166-supplementary.pdf]

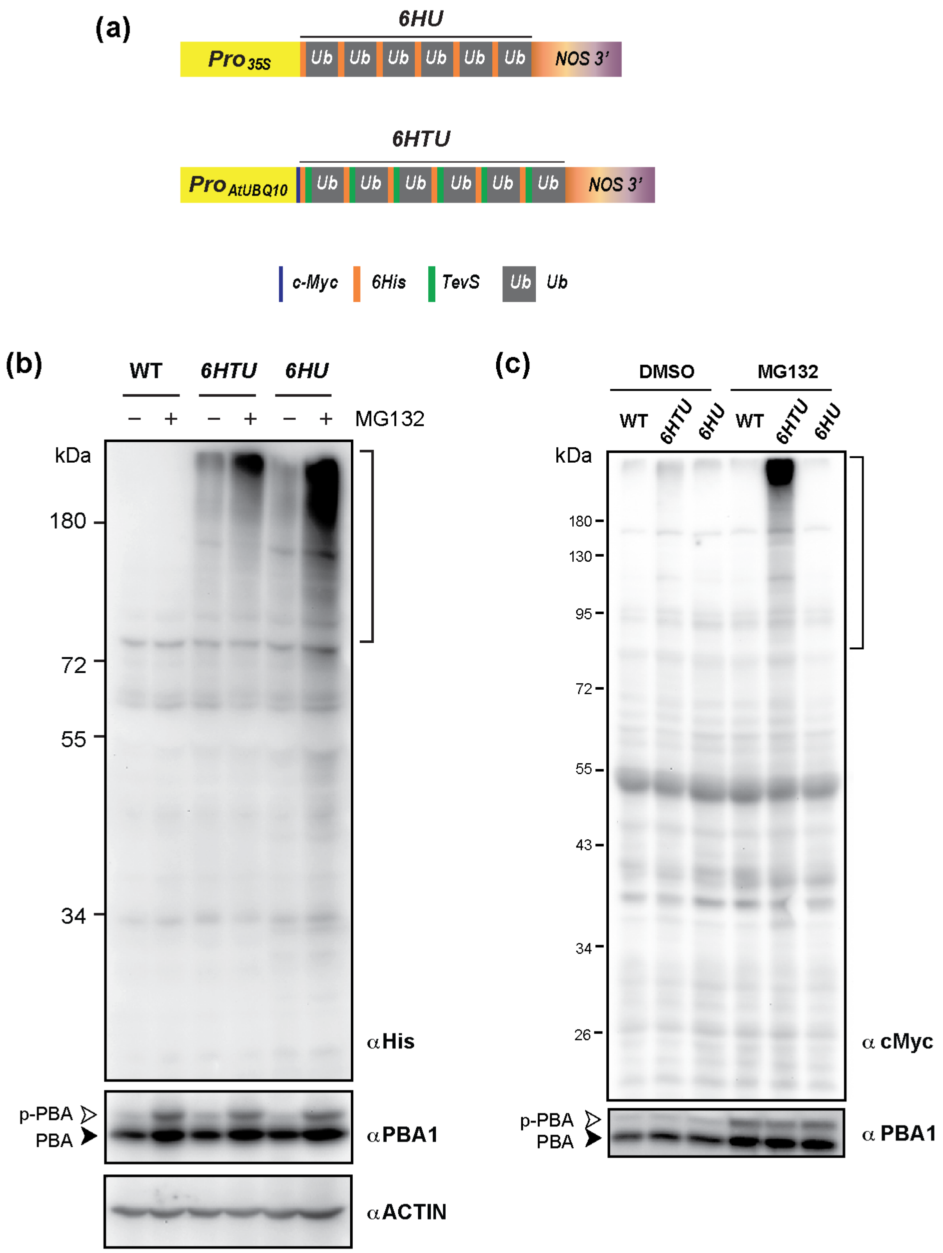

Figure S1. Comparison of HU and HTU conjugation suggests a strong and moderate expression of *6HU* and *6HTU* in 7-d-old LD-grown seedlings, respectively. (a) A schematic diagram showing the constructions of *6HU* and *6HTU*. *NOS 3'*: terminator of *Agrobacterium tumefaciens nopaline synthase* gene. *TEV*: *Tobacco Etch Virus protease cleavage site*. *c-Myc*: coding sequence of a c-Myc peptide added in-frame to the 5'-end of *6HTU*. (b) Immunoblot analysis indicates a strong and moderate expression of *6HU* and *6HTU* in 7-d-old LD-grown seedlings, respectively. The proteasome inhibition by MG132 is determined by an accumulation of the PBA1 precursor (open arrowhead) [41]. ACTIN is used to verify nearly equal loading of total protein. Bracket indicates high-molecular-mass HU or HTU conjugates. (c) Immunoblot analysis by an anti-cMyc antibody. The lack of a band matching the size of 6HTU and the HTU conjugates with a high molecular weight suggests a proper post-translational cleavage of mono HTU moieties from 6HTU by deubiquitylating proteases.

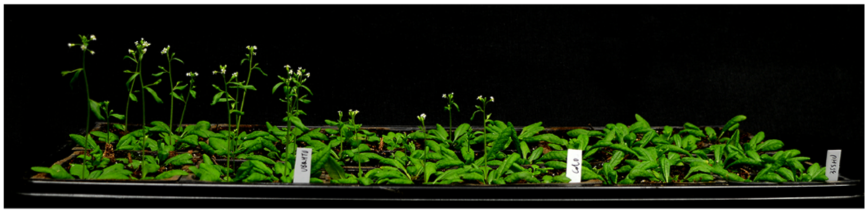

6HTU WT *6HU*

Figure S2. A large view of increasing and reducing growth vigor of *6HTU* and *6HU* plants, respectively, under a normal growth condition. Plants germinated from synchronized seeds were grown on the same tray under an LD-photoperiod for two weeks. Plants were photographed to show differential growth vigor among the three indicated genotypes.

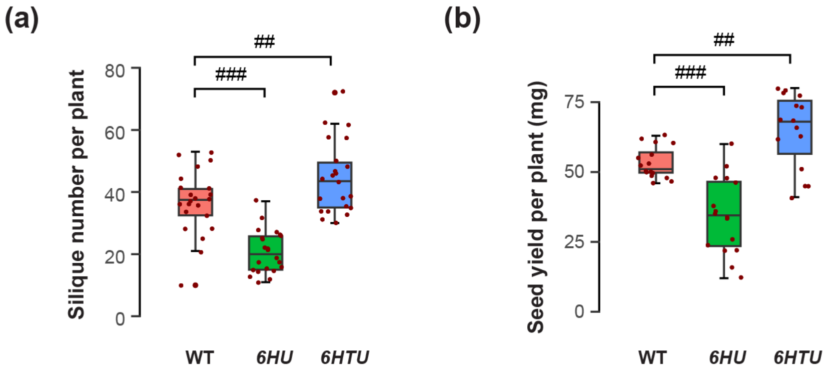

Figure S3. Moderate and strong expression levels of recombinant *UBQ* genes have an opposite impact on seed production per plant. Synchronized seeds were germinated and grown under an LD-photoperiod for both silique number and seed yield assays. (a) Opposite effects between moderate and strong expression of recombinant *UBQ* genes on the number of siliques developed per plant. At the ripening stage, individual plants were randomly selected from each indicated genotype and counted for the number of siliques developed. (b) Comparison of seed production per plant indicates an opposite effect between moderate and strong expression of recombinant *UBQ* genes. Ripened seeds were harvested carefully from randomly selected plants and dried in 1.5 mL tubes for 3 days in a 37°C incubator and acclimated at room temperature for 2 days before measurement. The data points of replicates and statistical analysis both in (a) and (b) are as described in Figure 1.

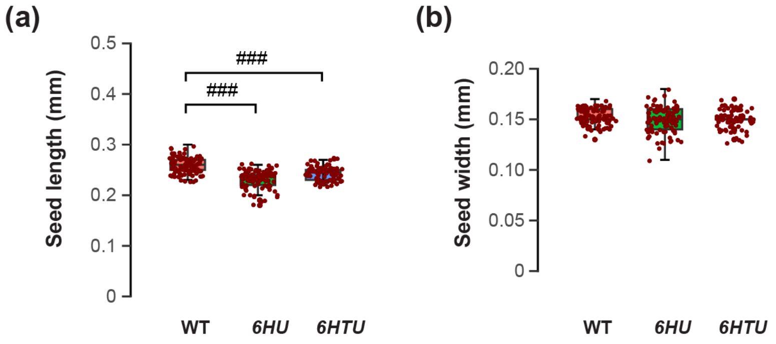

Figure S4. Reduced seed sizes in *6HU* and *6HTU* result from shortened length but not width. Ripened seeds from 48 plants were harvested, pooled, dried for 3 days in a 37°C incubator, and acclimated at room temperature for 2 days before measurement using Image J. (a) Shortened seed lengths in *6HU* and *6HTU* compared to WT. (b) No seed width difference observed among WT, *6HU*, and *6HTU*. The data points of replicates and statistical analysis both in (a) and (b) are as described in Figure 1.

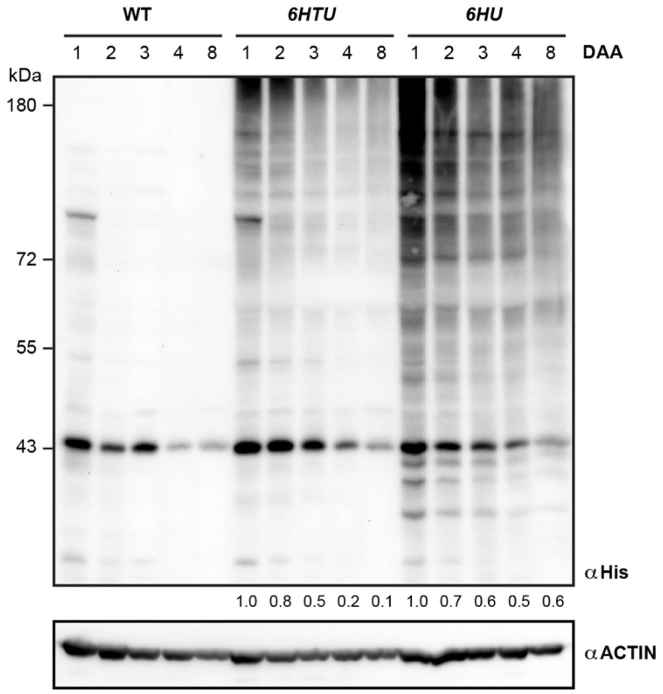

Figure S5. HU and HTU-conjugates showed different dynamic changes in early silique development. The same set of protein samples as in Figure 3b was assayed for HU and HTU conjugated proteins by anti-6His antibodies. The number indicated in each lane was calculated as in Figure 3b. The ACTIN blot was the same as in Figure 3b since both assays used the same set of samples.

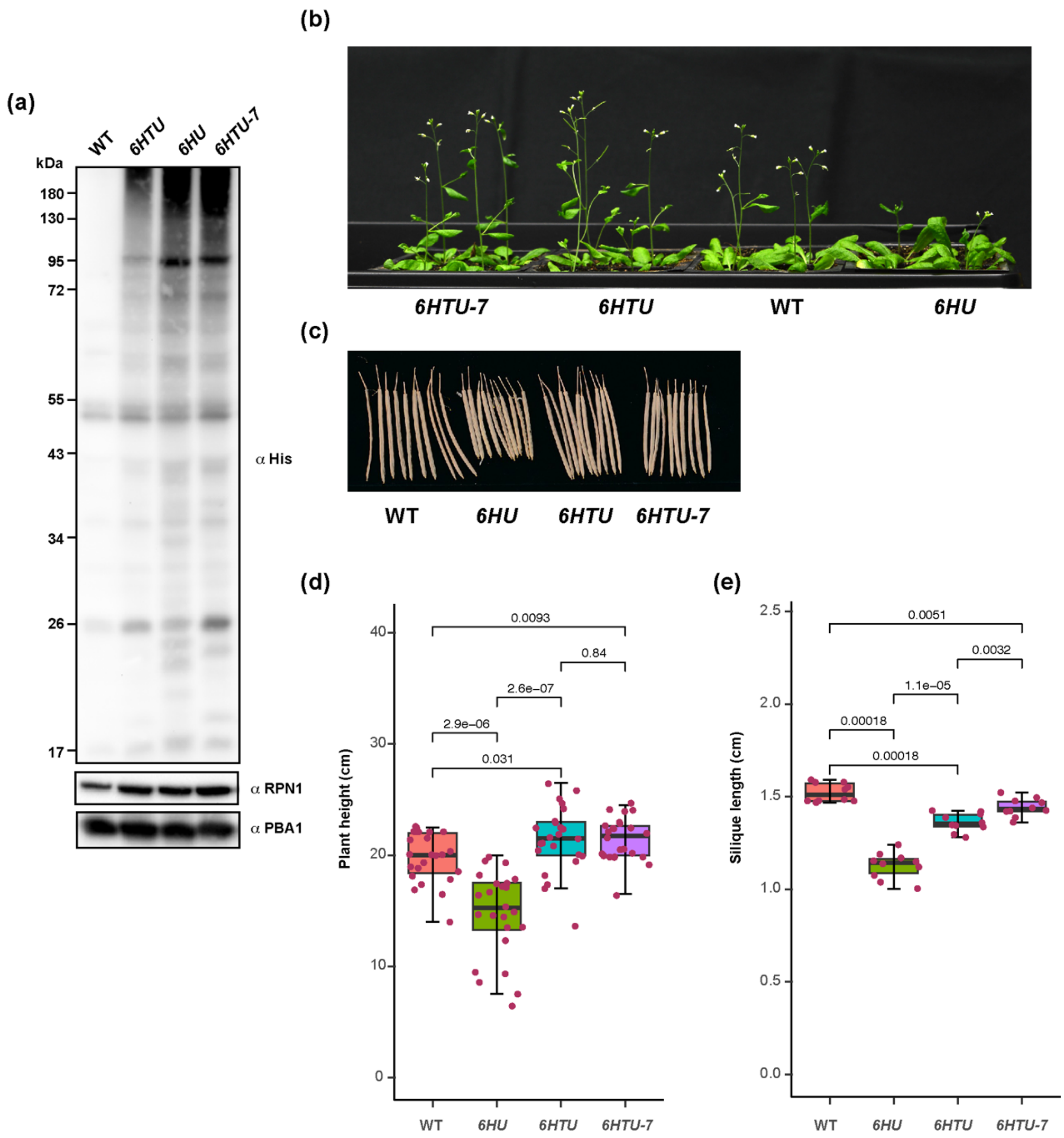

Figure S6. Increasing protein conjugation with HTU has a moderate effect on plant height and silique size of *6HTU*. (a) Immunoblotting analysis on 3-d-old seedlings identified a similar level of recombinant Ub conjugates in *6HTU-7* and *6HU*. Proteins conjugated with HTU and HU were detected using a monoclonal anti-6His antibody as in Figure S5. RPN1 and PBA1 were used as loading controls. (b) Representative plants at the bolting stage showing a higher growth vigor of two independent *6HTU* transgenic plants. The plant images of *6HTU*, *6HU*, and *WT* are the same as in Figure 1c. (c) Representative images showing shortened siliques developed from primary inflorescences of 6HU and 6HTU plants compared to WT. Siliques were selected and imaged as in Figure 2a. (d,e) Quantification of plant height and silique length as shown in (b) and (c), respectively. The data points of replicates in each boxplot are indicated with maroon dots. P values of each comparison were calculated using the Wilcoxon rank-sum test.
